# The role of macrophages in non-small cell lung cancer and advancements in 3D co-cultures

**DOI:** 10.7554/eLife.82998

**Published:** 2023-02-21

**Authors:** Katarína Balážová, Hans Clevers, Antonella FM Dost

**Affiliations:** 1 Hubrecht Institute for Developmental Biology and Stem Cell Research-KNAW & University Medical Centre Utrecht Utrecht Netherlands; 2 Oncode Institute, Hubrecht Institute-KNAW Utrecht Netherlands; 3 Roche Pharma Research and early Development Basel Switzerland; Tsinghua University China; King's College London United Kingdom

**Keywords:** macrophages, organoids, NSCLC, co-culture, lung cancer

## Abstract

Lung cancer (LC) is the leading cause of cancer-related deaths worldwide. Traditional therapeutic approaches such as chemotherapy or radiotherapy have provided only a marginal improvement in the treatment of lung carcinomas. Inhibitors targeting specific genetic aberrations present in non-small cell lung cancer (NSCLC), the most common subtype (85%), have improved the prognostic outlook, but due to the complexity of the LC mutational spectrum, only a fraction of patients benefit from these targeted molecular therapies. More recently, the realization that the immune infiltrate surrounding solid tumors can foster tumor-promoting inflammation has led to the development and implementation of anticancer immunotherapies in the clinic. In NSCLC, one of the most abundant leukocyte infiltrates is macrophages. These highly plastic phagocytes, which are part of the cellular repertoire of the innate immunity, can have a pivotal role in early NSCLC establishment, malignant progression, and tumor invasion. Emerging macrophage-targeting therapies have been focused on the re-differentiation of the macrophages toward an antitumorigenic phenotype, depletion of tumor-promoting macrophage subtypes, or combination therapies combining traditional cytotoxic treatments with immunotherapeutic agents. The most extensively used models employed for the exploration of NSCLC biology and therapy have been 2D cell lines and murine models. However, studying cancer immunology requires appropriately complex models. 3D platforms, including organoid models, are quickly advancing powerful tools to study immune cell-epithelial cell interactions within the tumor microenvironment. Co-cultures of immune cells along with NSCLC organoids allow for an in vitro observation of the tumor microenvironment dynamics closely resembling in vivo settings. Ultimately, the implementation of 3D organoid technology into tumor microenvironment-modeling platforms might facilitate the exploration of macrophage-targeted therapies in NSCLC immunotherapeutic research, thus establishing a new frontier in NSCLC treatment.

## Introduction

Lung cancer (LC) is the leading cause of cancer-associated death worldwide with an average survival being less than 5 years’ post-diagnosis (Ferlay et al., 2021). Mortality due to LC surpasses the combined mortalities caused by breast, colon, and prostate malignancies (Howlader, 2020). The two primary histological LC types are small cell lung cancer (SCLC), encompassing about 15% of all cases, and non-small cell lung cancer (NSCLC), the predominant subtype accounting for about 85% of cases (Conway et al., 2016; Li et al., 2020). Further sub-division of NSCLC includes lung adenocarcinoma (LUAD, ~40%), lung squamous cell carcinoma (LUSC, ~25%), and large cell carcinoma (~10%) (Arora et al., 2021). While surgical tumor resection has the greatest success therapeutically, the vast majority of patients (more than 80%) receive their diagnosis at the advanced stages of the disease, which makes surgical treatment difficult (Conway et al., 2016; Ettinger et al., 2017). In these cases, platinum-based chemotherapy and radiotherapy are used as the first line of treatment. However, the therapeutic effects of these traditional approaches are often modest and provide great discomfort to the patients due to their inherent toxicity (Conway et al., 2016; Kong et al., 2021; Sarode et al., 2020). With the recent but rapid advancement of next-generation sequencing methods, targeted therapies using small molecule inhibitors such as gefitinib or erlotinib (epidermal growth factor receptor [*EGFR*] tyrosine kinase inhibitors), among others, have been developed to exploit LC-specific mutations (Dearden et al., 2013; Chung, 2016). When compared to the traditional treatments, targeted therapy improved response rates but its utility has been limited to a fraction of LC cases, largely due to the complexity of the LC mutational landscape. The eventual acquisition of resistance of the tumor to targeted therapies has further hindered the improvement in survival rates, maintaining the average 5-year survival at around 18% (Conway et al., 2016; Kong et al., 2021).

Emerging evidence gathered over the past decade has repeatedly pointed out the importance of the tumor microenvironment (TME) in the development and progression of cancers. The involvement of the immune TME has even been highlighted as an emerging hallmark involved in multiple cancer types (Hanahan and Weinberg, 2011). The specific composition of the immune microenvironment surrounding the tumor can affect the prognosis, disease progression, and patient survival (Arora et al., 2021). Therapies aimed at modulating the immune microenvironment were long believed to be ineffective in LC as a limited response was observed with non-specific treatments (using interleukin 2 [IL-2] or interferon [IFN]). The immunogenicity of LC only became known following trials using immune checkpoint inhibitors targeting cytotoxic T lymphocyte-associated protein-4 and antiprogrammed cell death protein-1 (PD-1) (Rizvi et al., 2015; Lynch et al., 2012). The range of LC responses to immunotherapies remains variable, depending mostly on the mutational burden of the tumor and the subsequent neoantigen diversity which, together with a range of other factors (such as programmed death-ligand 1 [PD-L1] expression, interferon-γ [IFN-γ] signaling, and others) determine T cell reactivity against them (Schumacher and Schreiber, 2015; Hendriks et al., 2018; Hegde and Chen, 2020). Additionally, the changing mutational landscape of the lung tumor in response to these therapies along with evasion of immune surveillance often lead to the acquisition of chemoresistance (Kong et al., 2021).

Therefore, identification of the specific LC immune cell landscape and the tumorigenic processes associated with it have been crucial avenues unveiling promising treatment approaches for lung tumors (Kong et al., 2021; Sarode et al., 2020; Ruffell and Coussens, 2015). In particular, macrophages – phagocytic cells of the innate immunity – have garnered interest as the predominant cell type within the immune infiltrate in lung tumors. Macrophages are highly diverse and feature many phenotypes with different properties and functions. While their general presence within the immune infiltrate in cancer is mostly indicative of a negative prognosis, their effect on cancer progression appears subtype-dependent (Conway et al., 2016; Sarode et al., 2020; Cassetta and Pollard, 2018). As NSCLC represents the vast majority of LC cases, we focus on the implications of immunotherapeutic treatment options targeting the macrophage component in this highly deadly cancer. After briefly discussing the most recent knowledge of different macrophage subtypes and their potential roles in NSCLC development, we review the current scope of pre-clinical models for NSCLC, with a particular focus on 3D organoid-immune cell co-culture platforms. Such 3D platforms have been emerging rapidly over the past decades, and are designed to not only capture the highly complex macrophage-organoid dynamics but could also be a valuable resource for testing and developing new macrophage-targeted immunotherapies.

## I. Macrophage involvement in NSCLC development and progression

**Figure 1.**
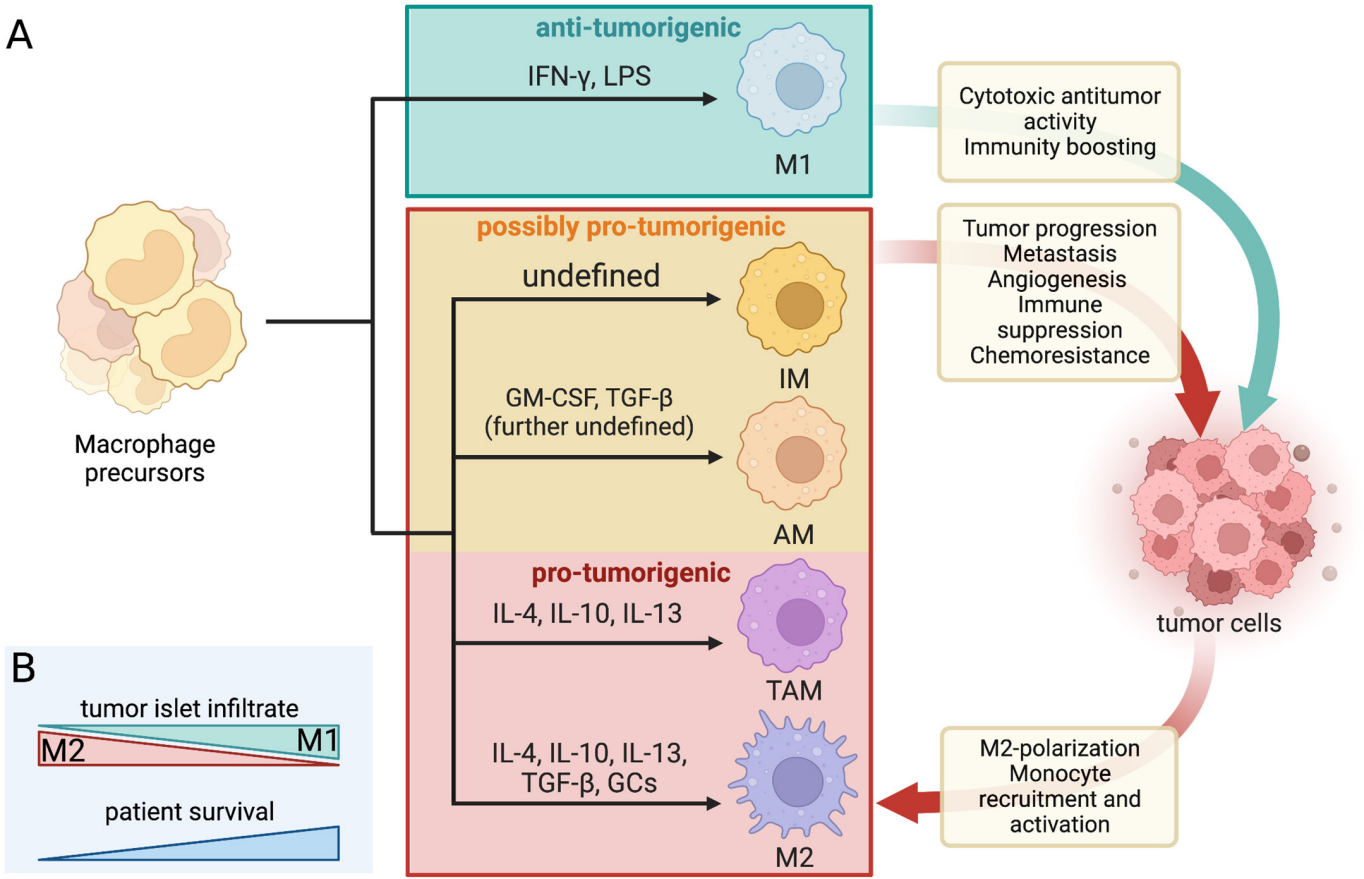
Dynamic crosstalk between pro- and antitumorigenic macrophage subtypes and tumor cells. (**A**) Macrophage differentiation is highly heterogenous and depends mostly on the environmental cues within their niche. The classical binary division includes the M1 (tumoricidal and pro-inflammatory) and M2 (tumor-supporting and antiinflammatory) polarization. The increasingly favored macrophage division considers macrophage diversity as a spectrum and includes tumor-associated macrophages (TAMs), alveolar macrophages (AMs), and interstitial macrophages (IMs) as separate subtypes with mostly pro-tumor properties in LC/NSCLC. The dynamic macrophage-tumor crosstalk within the TME results in different activation of the immune cells and confers a range of effects that can either aid the tumor development and progression or halt it. The included differentiation factors are the most represented within the existing literature. A range of other cues were found to contribute to macrophage differentiation but their effect is not yet well defined. The red and orange boxes mark all the macrophage subtypes that are generally considered pro-tumorigenic in LC/NSCLC settings and their associated effects on tumor cells (and vice versa). IL, interleukin; GM-CSF, granulocyte macrophage colony-stimulating factor; TGF, transforming growth factor; GCs, glucocorticoids; IFN, interferon; LPS, lipopolysaccharides. (**B**) The prognosis and survival of NSCLC patients are reflected by the macrophage infiltrate within the tumor islets. Greater M1 infiltrate generally indicates a favorable prognosis, while the predominance of M2 predicts reduced survival.

Cancer-associated inflammation is a process fueled by an arsenal of chronically activated immune cell subsets and their associated products within the TME (Coussens et al., 2013). One of the most highly represented leukocyte types within the LC TME are macrophages, with LUAD lesions featuring a particularly high abundance of these cells compared to other subtypes (Conway et al., 2016; Cassetta and Pollard, 2018; Kargl et al., 2017). Macrophages are widely distributed throughout the body. When innate barriers are breached by viruses or bacteria they ingest these pathogens and fight the infection. Under stable physiological conditions, macrophages contribute to processes such as wound healing, development of tissues, and maintenance of homeostasis. Their function and presence have also been implicated in a vast array of autoimmune disorders and tumorigenic processes (Conway et al., 2016; Aras and Zaidi, 2017; Zhu et al., 2020). They are either monocyte-derived and originate from the bone marrow or tissue-resident and originate from embryonic tissues such as fetal liver and the yolk sac. As such, they likely settle into their respective niches in successive waves during embryogenesis and further development. Depending on the anatomical site of settling they take on a tissue-specific identity which determines their transcriptional profile and specialization (Sreejit et al., 2020; Laviron and Boissonnas, 2019; Ginhoux and Guilliams, 2016; Blériot et al., 2020). Macrophages feature incredible phenotypic plasticity brought on by the specific tissue-dependent microenvironmental cues such as metabolite composition, the nature of phagocytosed particles, or the cellular constituents within the niche. After they receive specific environmental stimuli, they polarize and change the expression of their surface markers as well as alter their effector functions (Figure 1A; Aras and Zaidi, 2017; Sreejit et al., 2020; Rodero et al., 2015).

Created with BioRender.com

### Classically activated and alternative activated macrophages

The functional heterogeneity of macrophages often presents a nomenclature conundrum. The most well-established binary division of macrophage phenotypic states is based on an early observation of Stein et al., 1992, who recorded distinct phenotypes brought on by stimulation with interleukin 4 (IL-4) produced predominantly by T helper 2 cells (T_H_2), or IFN-γ produced primarily by T helper 1 and natural killer cells. IL-4 is an antiinflammatory cytokine that alternatively activates macrophages into a state of enhanced scavenging, restricted expression of major histocompatibility complex II (MHCII), and attenuated secretion of pro-inflammatory cytokines (in contrast to IFN-γ-stimulated macrophages) (Stein et al., 1992; Bissonnette et al., 2020; Chávez-Galán et al., 2015). The nomenclature for these opposing macrophage phenotypes was not established until the 2000s when their distinct metabolic profiles were identified, and they have been henceforth referred to as M1 (classically activated by IFN-γ and/or lipopolysaccharides [LPS]) and M2 (alternatively activated by IL-4, IL-10, IL-13, transforming growth factor β [TGF-β], and/or glucocorticoids) cells (Figure 1A; Conway et al., 2016; Arora et al., 2021; Chávez-Galán et al., 2015; Mills et al., 2000; Murray et al., 2014). The M1 phenotype is characterized by high expression of interleukin 1β (IL-1β), IL-6, tumor necrosis factor alfa (TNFα), as well as inducible nitric oxide synthase. Conversely, the M2 macrophages are unique for their high expression of arginase which blocks nitric oxide production through urea and ornithine synthesis. They also express high levels of IL-10 and TGF-β, cytokines with antiinflammatory functions (Arora et al., 2021; Bissonnette et al., 2020). Due to the secretion of primarily pro-inflammatory cytokines, M1 macrophages are generally antitumorigenic, while M2 macrophages seem to promote tumorigenicity via their antiinflammatory properties (Aras and Zaidi, 2017). The two macrophage polarization-driven subtypes vary by their transcriptomic profile, metabolism, surface markers, and cytokines produced (Sarode et al., 2020). A high overall density of macrophages within the NSCLC TME seems to be indicative of a favorable prognosis (Conway et al., 2016; O’Callaghan et al., 2010). However, the microanatomical distribution of the macrophage infiltrate and its polarization state are crucial for establishing a more accurate prognosis. As such, a regionally high infiltrate of M2-polarized macrophages in NSCLC tumor islets has a greater association with poor prognosis, while the predominant presence of M1 macrophages indicates increased chances of extended survival and favorable prognosis (Figure 1B; Ohri et al., 2009; Cao et al., 2019; Jackute et al., 2018; Sumitomo et al., 2019).

### Tumor-associated macrophages: separate subtype or M2-associated?

Nowadays, macrophage differentiation is increasingly considered to be a spectrum rather than two distinct phenotypes with opposing states of polarization. Tumor-associated macrophages (TAMs) reflect an activation state continuum and comprise a large portion of the TME infiltrate in solid tumors (Aras and Zaidi, 2017; Chávez-Galán et al., 2015). The microenvironment surrounding solid tumors is a hub for generating chemo-attractive molecules (such as C-C motif chemokine ligand 2 [CCL2] and colony-stimulating factor [CSF]) that recruit monocytes circulating in peripheral blood. Once at the tumor site, these inflammatory monocytes can be stimulated by a range of cytokines to differentiate into TAM phenotype cells (Figure 1A). The functional presence of TAMs is usually associated with the establishment of a tumor-supporting environment, thus relating to poor prognosis and serving as a potential prognostic marker (Cassetta and Pollard, 2018; Aras and Zaidi, 2017). While TAMs share some features with the M1/M2 macrophages, they have a distinct transcriptomic profile and are thus considered a separate macrophage subtype, although this distinction is often absent in the existing literature. Due to overlapping features, the macrophage subtypes have to be thoroughly characterized based on several markers. Clear identification of macrophage subtype associated with individual tumor types is a prerequisite for establishing a robust and accurate prognosis (Aras and Zaidi, 2017; Chávez-Galán et al., 2015; Murray et al., 2014).

Dynamic crosstalk between the cells of the tumor and the macrophage infiltrate is associated with tumorigenic processes such as invasion, metastasis, cancer progression, and angiogenesis (Aras and Zaidi, 2017; Almatroodi et al., 2016). LC cells maintaining stem cell-like properties via overexpression of Oct4 were found to secrete high levels of macrophage-CSF (M-CSF). M-CSF promotes TAM polarization toward the M2 phenotype, increasing tumor progression via enhanced cancer cell migration and metastasis (Lu et al., 2020). Persistent activation of nuclear factor κB (NF-κB) in LUAD epithelial cells enhances the pro-inflammatory nature of the TME, recruiting macrophages into the tumor site which in turn favors the development of metastatic foci (Stathopoulos et al., 2008). Similarly, elevated neddylation (a type of post-translational modification) fuels tumorigenesis in LC via enhanced expression of NF-κB in tumor cells. NF-κB transcriptionally activates CCL2, a potent chemokine that stimulates enhanced macrophage infiltration and subsequent TAM differentiation within the TME (Zhou et al., 2019). Similar to other solid tumors, NSCLC lesions feature a highly hypoxic TME which allows for downregulation of the complement component 9 (C9), the ultimate component of the innate complement system, in TAMs. This is accompanied by a phenotype transition from M1 into M2, thus leading to loss of anticancer functions of the M1 phenotype and tumor progression (Li et al., 2018). Moreover, high intratumoral heterogeneity in Kirsten rat sarcoma viral oncogene homolog (*Kras*)-driven LC allows for M2 polarization through circular RNA regulation, sustaining an immunosuppressive environment that favors metastasis and the acquisition of chemoresistance (Katopodi et al., 2021). NSCLC cell lines were also found to induce *Arginase-1* production in murine macrophages, enhancing their immunosuppressive M2-like phenotype (Park et al., 2022). Due to the bidirectional communication between the tumor cells and the immune infiltrate, macrophages can also contribute to establishing a pro-tumorigenic environment which may allow for evasion of immune surveillance at the tumor site (Hofman, 2020; Qiu et al., 2021). Accumulation of M2 TAMs in NSCLC stroma is associated with higher production of vascular endothelial growth factors A and C, thus supporting a pro-angiogenic and pro-lymphangiogenic environment adjacent to the tumor (Hwang et al., 2020). Moreover, secretion of factors such as TGF-β by TAMs (causing an increase in the sex-determining region Y-related high mobility group box 9 expression) contributes to the epithelial-mesenchymal transition (EMT) within NSCLC, promoting tissue remodeling and metastasis (Hofman, 2020; Zhang et al., 2017). Metastasis-promoting effects in LC are also elicited by M2-polarized macrophages via upregulation of αB-crystallin expression, inducing EMT and resulting in poor prognosis (Guo et al., 2019).

### Tissue-resident alveolar and interstitial macrophages

To add to the spectrum of phenotypically diverse macrophages, further macrophage subtypes distinct from the M1, M2, and TAM profiles have been identified (Figure 1A). Alveolar macrophages (AMs) normally function to maintain a steady state of the respiratory system by tempering the immune responses to avoid unnecessary inflammation and remove any physical debris that enters the airways (Aras and Zaidi, 2017; Bissonnette et al., 2020). Tissue-resident AMs are a lineage derived from the yolk sac and are capable of self-maintenance in adult tissues (Laviron and Boissonnas, 2019). Although their role in NSCLC tumorigenesis has been conflicting, they were recently found to associate with NSCLC lesions during early tumor formation (Almatroodi et al., 2014; Casanova-Acebes et al., 2021). AMs can contribute to early EMT via high expression of matrix metalloproteinases (*Mmp12*, *Mmp14*, *Adamdec1*) and support an immunosuppressive TME by recruiting regulatory T cells into the cancer site (through TGF-β, CCL17, and MHCII upregulation). This shields the tumor from cells of adaptive immunity. Depletion of CD169+ AMs very early in tumorigenesis (0–3 days) enhanced the antitumor environment (Casanova-Acebes et al., 2021; Li et al., 2021). CD169+ (also known as Siglec-1) is an antigen abundantly represented on macrophages found in the lung, liver, spleen, lymph nodes, and bone marrow. Information concerning the specific activation route of CD169+ macrophages is limited thus far, although their role seems to be less phagocytic and more immunoregulatory, depending on their localization (Aras and Zaidi, 2017; Chávez-Galán et al., 2015; Luo et al., 2021). Perhaps even less known tissue-resident macrophage subtype is the interstitial macrophage (IM). Under steady-state conditions, IMs are seemingly involved in the defense against airway allergies and other innate immune modulation (Liegeois et al., 2018). Phenotypically, studies using murine models show that there are at least two distinct populations of IMs mostly distinguished by Lyve1^high^MHCII^low^ or Lyve1^low^MHCII^high^ gene expression (Chakarov et al., 2019). IMs might also play a role in LC, as the presence of IMs and their IL-9-stimulated arginase production correlated with tumor growth in mouse lungs (Fu et al., 2022; Loyher et al., 2018). However, their low abundance (just 4% of lung monocytes) and a lack of defined markers in (human) tissues lead to scarcity of studies investigating their function, also in the context of NSCLC (Liegeois et al., 2018).

### Targeting macrophages as a therapeutic avenue in LC

The role of macrophages in LC remains elusive due to conflicting evidence associating macrophages with both positive and negative outcomes, but the focus is maintained on these innate cells as they make up the majority of the tumor immune infiltrate (Conway et al., 2016; Ruffell and Coussens, 2015). As such, a multitude of treatment options targeting the macrophage component within LC have been proposed. These range from anti-PD-1/PDL-1 therapy for NSCLC patients with abundant M2 infiltrate (Cao et al., 2019), promotion of C9 secretion in AMs to suspend NSCLC progression (Li et al., 2018), IL-9 signaling blockade (Fu et al., 2022), as well as skewing the M2 phenotype toward the M1 phenotype to elevate tumor-fighting properties within the TME (Conway et al., 2016; Cassetta and Pollard, 2018; Almatroodi et al., 2016; Li et al., 2018). In SCLC, the blockade of ‘do not eat me’ signals conveyed by CD47 expression on tumor cells and its interaction with signal regulatory protein alpha (SIRPα) on macrophages increased phagocytic activity of macrophages and inhibited tumor growth (Weiskopf et al., 2016; Lin et al., 2020). Preventative measures via depletion of a specific subset of macrophages located within the lung TME could also be taken (Casanova-Acebes et al., 2021). The acquisition of resistance to immunotherapies could be overcome by systematic identification of immune infiltrate in LC (Ruffell and Coussens, 2015; Horvath et al., 2020). Currently, the vast majority of therapies focused on the macrophage component in LC remain in the pre-clinical stages, as non-specific systemic targeting of TAMs proved to be detrimental to the health of the patients (Sedighzadeh et al., 2021; Kielbassa et al., 2019). Perhaps the most promising therapeutic results thus far have been observed in combination therapies, where the macrophage component is targeted concurrently with more conventional anticancer regimens such as checkpoint inhibitors, cytotoxic chemotherapies, or radiotherapy (Kong et al., 2021; Qiu et al., 2021; Sedighzadeh et al., 2021). Thus, the new frontier of immunotherapeutic treatment for LC is reliant on improved identification of macrophage micro-localization and more accurate subtyping to allow for targeted depletion or reprogramming of the tumor-promoting macrophage populations (Sarode et al., 2020; Sedighzadeh et al., 2021).

## II. Pre-clinical models for NSCLC

### Cell lines, patient-derived xenografts, and genetically engineered mouse models

Lungs serve as an interface between the outside and the internal structures of the body and are therefore constantly exposed to airborne materials. In particular, the inhalation of tobacco smoke and other air pollutants poses an increased risk for the development of LC as it drives the process of chronic inflammation and has mutagenic effects on the lung epithelium (Ettinger et al., 2010; Yoshida et al., 2020). To model LC dynamics connected to pollutant exposure and other mutagenic processes, a range of pre-clinical models has been used. 2D cell lines have been the standard tool in cancer research since the 1950s when the first immortalized cancer cell line was introduced (Fitzgerald et al., 2020). There are currently over 200 NSCLC cell lines available. Established NSCLC cell lines, such as A549 or PC-9 (both LUAD), have been used extensively as they are cost-effective, high-throughput, and easy to manipulate genetically (Hynds et al., 2021). However, cell lines fail to emulate the complexity of the TME and due to immortalized culturing spanning several decades, they do not retain mutational signatures present in the parental tumors. As such, cancer therapies evaluated using cell lines do not provide robust evidence of their efficacy in the clinic (Hynds et al., 2021; Ben-David et al., 2018). Cell cultures derived from primary NSCLC patient samples match the original tumor profile and are permissive for personalized drug testing, but are difficult to culture for prolonged periods. Nonetheless, cell lines remain widely favored in pre-clinical research due to their practicality (Hynds et al., 2021).

Implantation or subcutaneous injection of patient tumor material into a murine model (patient-derived xenograft [PDX]) can replicate the 3D structure of the tumor, allowing for tumor proliferation, vascularization, and the maintenance of the mutational profile of the original tumor for several passages. Thus, PDX are a superior tool for the prediction of therapy efficacy using novel drug regimes. The drawbacks of PDX include low efficiency of establishment (up to 60% failure rate), costly maintenance, and prolonged setups (up to 10 months), rendering these models especially impractical for personalized medicine (Li et al., 2020; Kim et al., 2019). Moreover, due to a mismatch in immune profiles between mice and humans, immune-deficient mice need to be used to avoid xenotransplant rejection. Using advanced murine models such as humanized mice with a reconstituted human hematopoietic system could partially overcome this issue, but their use is highly limited due to their immense costs (Fitzgerald et al., 2020; Graham, 2021).

The majority of NSCLC research has been done with genetically engineered mouse models (GEMMs), mostly due to the possibility of exploiting tumor-inducing as well as lineage-tracing methods (Hynds et al., 2021). The most widely used LUAD GEMMs feature oncogenic *Kras* mutations that model initial stages of LUAD, and *Trp53* alterations present in more than half of the NSCLC cases (Dearden et al., 2013; Meuwissen et al., 2001; Jackson et al., 2005; Jackson et al., 2001). Currently, there is greater availability of GEMMs that harbor other major genetic aberrations present in LUAD such as *EGFR* (Politi et al., 2006), *BRAF* (Dankort et al., 2007), and others. Although the development of LUSC GEMMs has been hampered by the absence of well-defined activating oncogenes, GEMMs of LUSC harboring a single (Ji et al., 2007; Xiao et al., 2013) or a combination of (Xu et al., 2014; Mukhopadhyay et al., 2014; Ferone et al., 2016) genetic alterations have been developed to elucidate driver alterations accompanying human LUSC establishment. Ultimately, GEMMs are limited by their differential biology to that of a human, particularly when TME composition is involved. Even though mouse models cannot fully capture tumor progression and establishment as would be present in human tissue, they remain a valuable tool in LC research (Fitzgerald et al., 2020; Hynds et al., 2021; Lancaster and Huch, 2019).

### Organoid approaches for cancer modeling

**Figure 2.**
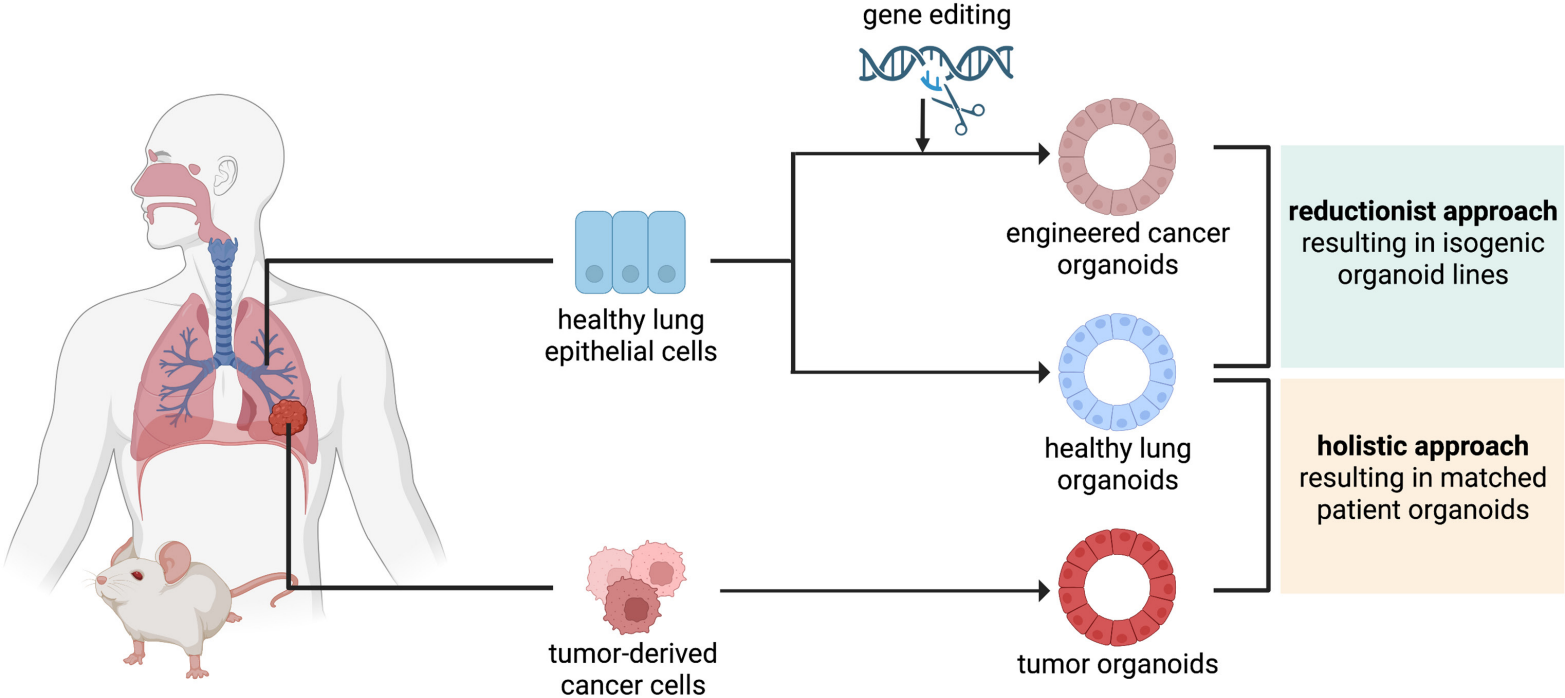
The reductionist and the holistic approaches to generate cancer organoids. In the reductionist approach, healthy lung epithelial cells are genetically engineered to carry non-small cell lung cancer (NSCLC) driver mutations. This approach works best if the cell of origin is known and culture conditions have already been established, to generate isogenic organoid lines. In the holistic approach, tumor-derived cancer cells are isolated and propagated as tumor organoids. If a healthy biopsy of the same patient can be obtained, this approach leads to matched patient organoids.

Over the recent years, lung organoids have become an increasingly popular tool for disease modeling and pre-clinical drug testing. Organoids are 3D structures derived from progenitor cells capable of self-assembly to reflect the structure, function, and genetic profile of the organ they are derived from, provided they are cultured in an environment emulating their stem cell niche in vivo (Sachs et al., 2019; Barkauskas et al., 2017). Organoids can be established from adult, embryonic, or induced pluripotent stem cells (iPSCs) (Clevers, 2016) however, for cancer modeling adult tissue is the preferred source. Human organoids complement current methods of pre-clinical testing and alleviate the burden of animal experimentation (Hofer and Lutolf, 2021; Werner et al., 2021). With the ultimate aim of creating organoid models that faithfully capture both the response of LC to drugs as well as allow for modeling of tumor progression, two general approaches can be taken: the holistic and the reductionist approach (Figure 2).

Created with BioRender.com

In the more widespread holistic approach, organoids are directly derived from lung tumor tissues of a patient or a model animal. The primary aim of using tumor samples from patients is to create a platform for drug testing (Hofer and Lutolf, 2021). Organoids derived from NSCLC patient material (patient-derived organoids [PDOs]) recapitulated the parental tumor histology and mutational profile. The long-term expansion and relatively small amount of human material required for their establishment have allowed for high-throughput drug screening, paving the way for personalized medicine (Sachs et al., 2019). Tumor organoids can also be established from PDX models (xenograft-derived organoids [XDOs]). Established PDOs and XDOs represented the two major NSCLC subtypes (LUAD and LUSC) and reflected the histology and tumorigenicity as well as drug sensitivity of their parental tumors. Due to the versatility of culturing under both short- and long-term conditions, they emerged as valuable platforms for biological experimentation and drug testing (Shi et al., 2020). To encompass the genetic diversity of LC, great effort has been invested in the creation of large-scale organoid biobanks containing hundreds of patient samples representative of all the LC subtypes. The PDOs are tested for genetic similarity with the original tumors, their histological structure, and drug sensitivities (Li et al., 2020; Kim et al., 2019; Sachs et al., 2019; Kim et al., 2021). With such a large undertaking, high-throughput screening has become an increasingly practical and efficient method of pre-clinical testing using chemotherapeutics and targeted molecular agents. PDOs can also be used to model advanced-stage LUAD. When treated with targeted anticancer therapies, the response of advanced LUAD PDOs reflected the responses and progression-free survival of the patients they originated from. Such PDOs can thus be used to model therapeutic responses to novel drugs targeting different tumor vulnerabilities or a combination of pre-existing therapeutics (Kim et al., 2021). Combination therapies using two or more targeted inhibitors (such as trametinib as MEK inhibitor and dabrafenib or vemurafenib as B-Raf inhibitor) are becoming increasingly investigated for their resistance-preventing properties as well as their antitumor efficacy (Park et al., 2013; Planchard et al., 2016; Joshi et al., 2015). Ideally, healthy patient-matched tissue should be used to generate healthy organoids to serve as a control. Long-term culturing conditions for organoids derived from airway cells are well established (Lancaster and Huch, 2019; Sachs et al., 2019). More recently, conditions for culturing adult alveolar organoids have been published, greatly advancing the lung organoid tool box (Konishi et al., 2022; Katsura et al., 2020; Youk et al., 2020).

In the reductionist approach, a healthy tissue sample is engineered with mutations that emulate the genetic alterations accompanying tumor initiation and progression. For this approach, the cell of origin should be known and culturing conditions should have been established. This approach has been extensively employed to model how genetic alterations within the intestine drive colorectal cancer (CRC) and human melanoma progression (Matano et al., 2015; Drost et al., 2015; Hodis et al., 2022). For CRC modeling, human small intestinal stem cell organoid cultures were modified using clustered regularly interspersed short palindromic repeat (CRISPR)/CRISPR-associated protein 9 genome editing (Cas9) system to introduce four defined CRC driver mutations (KRAS^G12D^, loss of APC, P53, and SMAD4) (Matano et al., 2015; Drost et al., 2015). The mutations were introduced in a defined sequence to recapitulate the adenoma to carcinoma progression as observed in human CRC samples (Vogelstein et al., 1988). Consequently, the engineered organoids acquired the ability to grow independent of factors usually required by the cell of origin, a feature characteristic for patient-derived CRC organoids (Matano et al., 2015; Drost et al., 2015). Similar CRISPR/Cas9-based approach was used to study genotype-phenotype associations in human melanoma. To achieve this, up to six sequential mutations (*CDKN2A, BRAF^V600E^, TERT, PTEN, TP53,* and *APC*) were introduced into healthy human melanocytes. The mutant cells partially recapitulated several defining characteristics of melanoma pathogenesis. Interestingly, depending on the combination of mutations, the immune TME underwent changes in the form of variable neutrophil abundance or genotype-specific gene expression profiles of macrophages. Due to the promising results of employing the reductionist approach in melanoma, the authors suggested its future expansion into a 3D skin organoid model (Hodis et al., 2022). Organoid models employing a bottom-up approach have been valuable for informing about the process of tumorigenesis. However, they have remained largely unexplored in the context of LC. Two recent studies have described methods to introduce LUAD driver mutations to murine cells in vitro, culturing them as organoids subsequently. However, both studies made use of already existing LC GEMMs, restricting the versatility of this system to already available mouse models (Dost et al., 2020; Naranjo et al., 2022). LUSC shares characteristics and markers with basal cells, which are present in airway organoids; LUAD is thought to arise from alveolar cells (Sachs et al., 2019; Sainz de Aja et al., 2021; Hanna and Onaitis, 2013). Even though culturing conditions for these cell types have been established in recent years, to this date there is no publication describing the introduction of LC driver mutations into healthy human lung organoids. Modeling lung tumor progression from the very initial stages using isogenic organoid lines would provide valuable information about molecular events that contribute to early carcinogenesis with aims to prevent tumor progression as well as to identify targets for LC treatment (Hynds et al., 2021; Dost et al., 2020).

### Outstanding organoid challenges

Despite these recent developments, organoids have certain limitations. In particular, the establishment of pure NSCLC organoids has its challenges. NSCLC organoids are frequently outgrown by non-cancerous cells and the establishment of pure organoids has a low success rate of 17%, especially when sourced from intrapulmonary tumor lesions (Dijkstra et al., 2020). Limited availability of pure tumor organoids limits their use in clinical research. It is recommended that the NSCLC organoid purity is thoroughly tested with immunostaining methods combined with traditionally used histo-morphological identification to distinguish cancer from normal lung organoids. Sourcing of NSCLC cells from metastatic lesions to enhance establishment rate is also possible, although this limits the modeling of primary cancers (Werner et al., 2021; Dijkstra et al., 2020). Increased efficiency of organoid establishment and prevention of over-passaging are needed to prevent excessive deviation from original tumor histopathology in personalized drug screens (Werner et al., 2021; Shi et al., 2020). Moreover, LC develops in the context of its immune TME which interacts strongly with the tumor cells, creating a highly complex feedback system that could result in either pro- or antitumorigenic effects. As such, epithelium-derived cancer organoids lack the capacity to fully recapitulate tumor progression as supported by the various components in the TME (Kim et al., 2019). However, our understanding of cancer biology is rapidly expanding. With organoid models becoming more readily available, the technological possibilities of incorporating TME elements within organoid models are rapidly widening as well (Fitzgerald et al., 2020; Bar-Ephraim et al., 2020).

## III. Possible implications of 3D NSCLC-macrophage co-culture

There is a high abundance of immune cells orchestrating a variety of protective functions within the lung epithelium. The lung epithelial cells secrete molecules to signal monocytes surveilling the blood periphery to either maintain a homeostatic state or to induce monocyte maturation into macrophages or dendritic cells during a state of infection (Rodero et al., 2015; Jose et al., 2020). Apart from their protective functions, immune cells can contribute to the establishment of a tumor-promoting environment, designating them as possible targets for pharmacological and cellular immunotherapies (Linde et al., 2012; Yuki et al., 2020). Up until now, the most extensively used in vitro platforms for observing immune-tumor cell interactions in NSCLC have been 2D co-culture models (Yuan et al., 2019). 2D co-culturing is highly accessible and easily modulated, but it only poorly imitates the conditions in vivo. As a result, the efficacy of cancer therapies tested in 2D co-cultures can be largely overestimated (Hynds et al., 2021; Majety et al., 2015). The ongoing advancements in tumor organoids have ensured wider availability of more complex 3D model systems which could gradually displace 2D co-culturing. Thus far, the organoid breakthrough has successfully advanced immunological anticancer research, enabled the exploration of cancer immunology, and facilitated the design of personalized immunotherapies (Yuki et al., 2020; Grönholm et al., 2021; Shamir and Ewald, 2014).

### Organoid co-culture with immune cells

**Figure 3.**
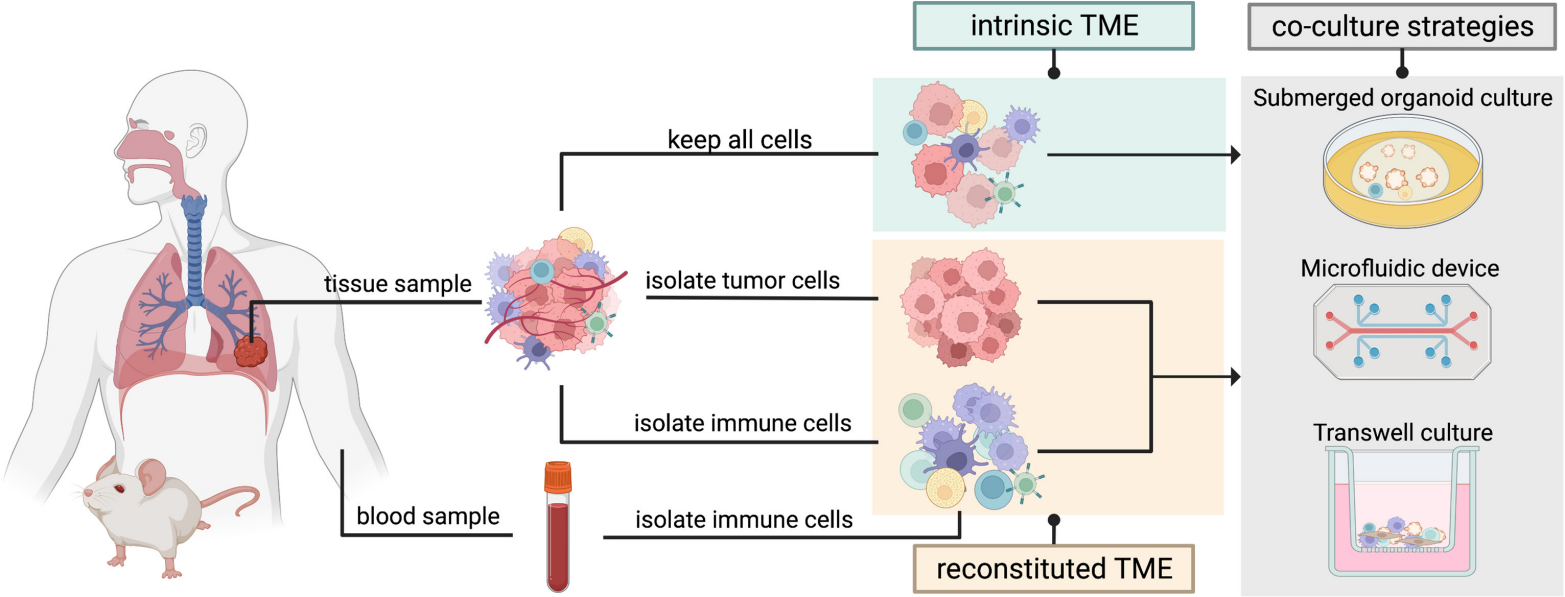
Two organoid co-culture approaches incorporating immune components of the tumor microenvironment (TME). In the intrinsic TME approach the non-epithelial cells of the TME are conserved along with the tumor cells. In the reconstituted TME approach tumor cells are isolated from the initial tumor biopsy and cultured separately from the immune cells. Immune cells sourced from peripheral blood or from the same tissue sample can then be added to the co-culture to reconstitute the TME in a controlled manner. Several methods can be used for immune cell-tumor organoid co-cultures: submerged organoid culture, micro-fluidic devices (organ-on-a-chip), or transwell cultures.

There are generally two approaches when considering a co-culture of immune cells with cancer organoids (Figure 3). The first approach preserves the intrinsic TME including immune components and other cells of non-epithelial origin from the tumor (PDO or murine) biopsy which are then cultured along with the epithelial tumor cells in submerged extracellular matrix domes, microfluidic devices such as organs-on-a-chip, or in transwell cultures that can mimic the air-liquid interface (ALI). While retention of intrinsic TME maintains a great range of cellular diversity, the culturing timeframe is restricted due to the difficulty of providing suitable culturing conditions for an array of immune, epithelial, and stromal cells (Yuki et al., 2020; Neal et al., 2018). With passaging, epithelial cells get enriched for and stromal cells are not maintained in the cultures.

Created with BioRender.com.

The second approach involves culturing organoids stripped of any non-tumor cells, with the immune cells sourced separately from the tissue or from a sample of peripheral blood (Bar-Ephraim et al., 2020; Yuki et al., 2020). While certain immune cells such as peripheral blood lymphocytes need to be human leukocyte antigen (HLA)-matched with the epithelial tumor cells, innate immune cells such as macrophages can be sourced from non-HLA-matched donor blood. Culturing conditions should be optimized for the propagation of the immune cell of interest. With this approach, the immune cells and the tumor cells can be combined in a co-culture in a controlled manner (Dijkstra et al., 2018). Overall, the first co-culture approach maintains the in vivo composition of the original tumor, thus allowing for testing of immunotherapeutic approaches (e.g. immune checkpoint inhibitors), while the second approach supports long-term culturing and an in-depth investigation of cell-cell interactions (Bar-Ephraim et al., 2020; Yuki et al., 2020; Zahmatkesh et al., 2021).

### Co-cultures with macrophages in cancer settings

The advent of more complex in vitro 3D co-culture systems has enabled the monitoring of TAMs in biologically relevant setups with various types of tumors. Although there is a lack of such research in NSCLC settings, observations from other cancers might provide valuable insight into TAM-tumor dynamics in 3D settings. In 2012, Linde and colleagues incorporated macrophages into the collagen matrix of organotypic human and murine skin squamous cell carcinoma (SCC) co-cultures. The methodology involved the growth of SCC cells atop fibroblast-like collagen-I gel (emulating the fibroblast component). Under steady-state conditions, direct keratinocyte interaction with fibroblasts is required to allow for growth and differentiation of the epithelial cells, but in cancer settings fibroblasts create a platform supportive of tumor growth. This co-culture method enabled for mixing of a cellular component matrix to retain the physiological context of the tumor, while also permitting the integration of bone marrow or peripheral blood-derived macrophages into the system. The addition of recombinant LPS or IFN-γ into the growth media resulted in strong macrophage polarization toward the inflammatory M1 phenotype. In contrast, stimulation of the organotypic co-culture system with IL-4 was found to support macrophage polarization toward the M2 phenotype, resulting in the breakdown of both the collagen and the basement membrane via the release of MMPs. Interestingly, prolonged co-culturing of the organotypic tumor with macrophages devoid of any exogenous stimulus resulted in a spontaneous M2 polarization. This polarization occurred independently of IL-4, as no IL-4 was detected in the stimulus-free cultures by ELISA. The proposed fibroblast-tumor-macrophage co-culture setup likely simulates in vivo-like tumor-TME dynamics and could be used for extensive studies of macrophage function in a cancer context (Linde et al., 2012).

Similar to LC, mammary adenocarcinomas are characterized by a high abundance of macrophages within the neoplastic tissue, along with increased infiltration of cells of the adaptive immunity (B and T lymphocytes) (DeNardo et al., 2009; Ruffell et al., 2012). Using organoids derived from a transgenic *MMTV-PyMT* mammary adenocarcinoma murine model, DeNardo et al., 2009, investigated the roles of CD4^+^ T lymphocytes and macrophages in tumor progression and metastasis in malignant epithelial tissues. The co-culture of *PyMT-*derived invasive organoids and TAMs revealed that TAMs (which were immunofluorescently labeled) predominantly localize along the invasive front of the organoid structures. Supplementation of this co-culture with IL-4 or IL-13 (T_H_2-derived cytokines) resulted in the amplification of invasive properties as well as disruption of the organoids in an IL/TAM dose-dependent manner. Conversely, the addition of LPS, IFN-γ, or IL-10 resulted in enhanced organoid stability. A ‘triculture’ setup of the *PyMT-*derived organoids, CD4^+^ T effector cells directly derived from *PyMT* mammary tumors and TAMs without exogenous stimulation revealed the M2-like TAM phenotype was promoted by higher IL-4 expression in CD4^+^ T cells. This induction of invasive properties was absent in carcinomas with depleted CD4^+^ T cell component or in the presence of M1-activating and immunoregulatory cytokines (DeNardo et al., 2009).

3D spheroid models aimed at engineering the TME were also employed to provide insight into macrophage association with malignant tissues. Spheroids are randomly distributed aggregates of cancer cells that are unable of self-assembly or regeneration but are useful models of immune interactions (Fiorini et al., 2020). A hanging-drop hetero-spheroid model of ovarian cancer stem cells with integrated macrophage component revealed an upregulation of the M2-polarization marker driven by IL-10 and WNT release from the cancer stem cells. Reciprocal WNT signaling of the M2 macrophages then maintained stemness of the cancer stem cells. The collaborative positive feedback signaling of both the tumor cells and macrophages resulted in an immunosuppressive environment supportive of enhanced cancer aggressiveness and the acquisition of chemoresistance (Raghavan et al., 2019). Overall, these findings could be exploited therapeutically as they identified the link between adaptive and innate immunity-associated components (both cellular and molecular) in malignancies of the breast and ovaries (DeNardo et al., 2009; Raghavan et al., 2019). As with any newly established methods, limitations are still present. In particular, while PDO co-systems reflect therapeutic vulnerabilities of parental tumor tissues, it is unclear whether short-term responses of organoids in culture would capture long-term treatment prognosis in patients and how this would be reflected in personalized immunotherapies (Bar-Ephraim et al., 2020; Yuki et al., 2020; Grönholm et al., 2021). Nonetheless, macrophage incorporation into a 3D system is gradually becoming a promising research avenue for an improved investigation of cancer dynamics, cell-cell crosstalk, as well as testing of anticancer therapeutic approaches.

### Co-cultures with macrophages in non-cancerous lung settings

Even though macrophage-organoid co-cultures have not yet been studied extensively in the context of LC, a great amount of investigative effort has been invested into co-cultures of lung organoids with macrophages in cancer-unrelated contexts. Such research can still provide valuable insight into the methodological approaches available and the technology employed for capturing cell-cell TME crosstalk. As such, a 2020 study by Jose et al., 2020 aimed to address the ability of iPSC-derived lung organoids to emulate human immunocompetent mucosal tissues. The incorporation of monocytes into lung organoid culture resulted in their migration toward the lung epithelium while supporting their maturation, as confirmed by bulk RNA sequencing. The attraction of host macrophages was observable under homeostatic conditions as well as during inflammation, suggesting 3D organoids could be used to explore a range of disease states (Jose et al., 2020).

To investigate the role of macrophages in lung development and tissue repair post-injury, Vazquez-Armendariz et al., 2020, developed a 3D bronchioalveolar lung organoid (BALO) model derived from murine lung cells . They were generated from bronchioalveolar stem cells, progenitors of various subsets of pulmonary epithelial cells (club cells, alveolar epithelial cells 1/2, and ciliated cells) in normal lung and precursors of LUAD (Kim et al., 2005; Salwig et al., 2019). BALOs were maintained alongside resident mesenchymal cells (Vazquez-Armendariz et al., 2020). As tissue-resident macrophages represent a crucial component of early embryonic lung development, integrating them in the BALO model system revealed the cooperation of tissue-resident macrophages with the epithelium to guide cell differentiation and downregulation of the inflammatory response. In contrast, the inflammatory response was enhanced by these macrophages upon the introduction of the influenza virus into the system. Macrophages were incorporated into the BALO model system via microinjection to ensure engraftment of macrophages facing the apical side of epithelial cells. The transcriptomic analysis using single-cell RNA sequencing revealed the downregulation of proliferation and inflammation-associated genes and the upregulation of cell clusters responsible for cell differentiation under steady-state conditions. The organoid-macrophage crosstalk was visualized using electron microscopy via the observation of microvillous protrusions (Vazquez-Armendariz et al., 2020). Taken together, the engraftment of tissue-resident macrophages into organoid cultures provides a platform for the investigation of detailed functions and interactions of the epithelial cells with cells of the innate immune system expandable to a variety of lung pathologies.

### Transwell cultures

Transwell setups feature a permeable membrane that is available with different pore sizes, which allows for the seeding of different cell types in different compartments that are connected through micropores. With this system, epithelial cells can be seeded on top of the membrane, while (conditioned) media and/or immune cells can be added to the bottom compartment, modeling the basolateral positioning of the immune cells in most epithelia (Guo et al., 2019; Bar-Ephraim et al., 2020; Lacroix et al., 2018). As one example, Noel et al., 2017, used this system to culture enteroid cells together with macrophages that were attached to the bottom compartment-facing side of the membrane. They traced the epithelial-immune cell crosstalk via changes in the cell morphology including ruffling of edges in macrophages and increased cell height of the epithelial cells. This allowed for observations of morphological and cytokine changes in both cell types, and the co-culture model was deemed reproducible for modeling gut physiology (Noel et al., 2017). In the lung field, transwells are often used as ALI systems. Lung cells derived from human bronchial epithelial cell cultures, organoids, or other sources first expand in 2D on top of the membrane in a submerged state. To induce differentiation of the cells to a pseudostratified epithelium, the liquid is then removed from the apical side of the epithelial cell layer so that the cells are exposed to air, mimicking the conditions of the respiratory tract (Pezzulo et al., 2011; Ghosh et al., 2020). Using these physiologically relevant ALI cultures, a variety of TME cellular components could be introduced into this system in the future.

### A microfluidic device: lung-on-a-chip

Cancer model systems can now be expanded into previously unforeseen levels of complexity. Organoid-complementary ‘on-a-chip’ microfluidic devices serve as improved model systems allowing for advanced and long-term modeling of the complex lung microenvironment (Figure 3; Hofer and Lutolf, 2021; Evans and Lee, 2020). Initially devised by Huh and colleagues in 2010, organs-on-a-chip are biomimetic microsystems that reconstitute different tissue elements as present in various in vivo organ systems, such as the lung (Huh et al., 2010). This biomimetic method can also be successfully utilized to model NSCLC progression, establish NSCLC responses to anticancer drug treatments, as well as to analyze persister cells to uncover mechanisms underlying tumor dormancy in LC (Hassell et al., 2017). Despite recent advancements in immunological research implicating immune cells as key players in various stages of tumorigenesis, immune systems-on-a-chip remain an underrepresented research avenue. The extensive amenability of these microfluidic devices renders them ideal for the study of immune cell migration toward cancer tissues in a manner otherwise unreproducible with other model systems. Initial immune systems-on-a-chip setups preferentially focus on cells of the innate immune system (such as macrophages) as the innate immune responses are faster compared to delayed adaptive immunity (Guo et al., 2019; Grönholm et al., 2021; Polini et al., 2019). The rapid advancements of the on-a-chip devices might eventually lead to the implementation of complex NSCLC model systems into anticancer research, paving the way for more advanced therapies for lung carcinomas.

## Discussion and future directions

The vast majority of LC cases are attributable to the effects of smoking and air pollution. The repetitive tissue damage caused by tobacco inhalation leads to high levels of immune cell infiltration, causing widespread tumor-promoting inflammation. Smoking also induces specific mutations within the lung epithelium, which can then generate a large number of neoantigens (Schumacher and Schreiber, 2015; Alexandrov et al., 2013). A high neoantigen load can be exploited and targeted by personalized immunotherapies, such as PD-1/PDL-1 blockade, focused on the amplification of T cell reactivity against cancer cells (Ye et al., 2021). However, the large reliance of immune checkpoint inhibitors on the neoantigen load limits their utility to a subgroup of NSCLC patients, leading the search for NSCLC therapies toward the immune landscape of the TME.

Macrophages, which are highly abundant within the innate immune infiltrate of the NSCLC TME, lead a dynamic crosstalk with the epithelial cells of the NSCLC tumors and contribute to NSCLC establishment, tumor progression, metastasis, angiogenesis, immunosuppression, and the acquisition of chemoresistance. However, high macrophage phenotypic plasticity, uncertainties concerning their origins, and the impact of niche-dependent signaling on their phenotype lead to persistent confusion in deciphering their specific functions within NSCLC lesions. As systematic depletion of macrophages causes a lot of side effects in patients, continuous research efforts have been aimed at unveiling markers specific for tumor-supporting macrophage subtypes. For instance, the centrally positioned AMs residing within the lung tissue have been assigned conflicting roles in NSCLC. The most recent efforts identified that CD169+ tissue-resident AMs in NSCLC tissues contribute to enhanced tumor growth and attenuate T cell-mediated antitumor response, elucidating their depletion as an emerging treatment for early NSCLC lesions (Casanova-Acebes et al., 2021). Because AMs reside in the alveolar space and are therefore present on the apical side of the epithelium, regular basal-out polarity organoid co-cultures with AMs would not accurately model the AM-epithelial cell interactions. Apical-out airway organoid cultures or transwell systems with AMs seeded on top of the epithelial cell layer would therefore be superior methods to study these interactions in vitro (Stroulios et al., 2022). Indeed, AMs are one of the first immune cells to encounter a newly transformed LC cell in vivo. Therefore, co-culturing AMs with early-stage LC cells or with engineered cancer using the above-mentioned culturing setups could deliver valuable insights into AM-cancer cell crosstalk.

As more knowledge is gathered concerning the specific cues leading to M1 and M2 polarization, therapies such as IFN-γ supplementation could lead to a re-differentiation of M2 macrophages toward the tumoricidal M1 phenotype, thus preventing cancer progression. Alternatively, the treatment focus could shift toward signaling molecules that enable cell-cell crosstalk. Targeting CCL2 or CSF released from the tumor cells could block the recruitment of monocytes toward the NSCLC lesions, thus preventing TAM infiltration and differentiation (Sedighzadeh et al., 2021). Similarly, the neutralization of IL-4-induced pro-TAM signaling may be a potent method of enhancing cytotoxic properties of the innate and adaptive immunity cells, allowing the immune system to efficiently target tumors (DeNardo et al., 2009). The disruption of the phagocytosis-suppressing CD47-SIRPα axis could promote macrophage-induced killing of LC cells (Weiskopf et al., 2016; Lin et al., 2020). The therapeutic agents could also be administered in combination to achieve a synergistic anticancer effect. Finally, it is important to note that continuous exposure to tobacco diminishes therapeutic outcomes, making smoking cessation a crucial part of any LC therapy (Cataldo et al., 2010).

The ever-expanding armamentarium of therapeutic regimens requires adequate testing platforms. The many models available for NSCLC research have advanced our understanding of LC biology and facilitated the implementation of numerous therapeutic regimens such as the combination of MEK and B-Raf inhibitor into the clinic (Planchard et al., 2016; Joshi et al., 2015). As the model systems are gradually shifting from 2D into 3D settings, 3D organoid models derived from either healthy or NSCLC tissues are continuously improved to faithfully capture features specific to human lung malignancies. To start, macrophages could be systematically incorporated into NSCLC organoids derived from wild-type tissues that have been genetically engineered to emulate NSCLC driver alterations. This approach could identify the specific macrophage-tumor crosstalk accompanying the initial stages of tumor establishment. It would be reasonable to focus these initial co-culturing efforts on LUAD organoids, as LUAD lesions are the most represented NSLSC subtype where high macrophages infiltrate might be of great relevance. However, it is important to recognize that with this stripped-down co-culturing method, the signaling from other immune or stromal cells toward the macrophages or the tumor epithelium itself would be absent.

The relationship between the development of immunotherapies and an advancement in their application in 3D settings is already becoming apparent. A potentially promising novel approach in TAM-focused immunotherapy is emerging in the form of chimeric antigen receptor-modified macrophages (CAR-M). The currently developed CAR-M therapies are primarily (but not exclusively) focused on the enhancement of the macrophage phagocytic activity against cells in solid tumors. In this way, CAR-M therapy aims to address shortcomings of its predecessor – the CAR-T cell therapy – which has been mostly successful in hematological tumors (Abdin et al., 2021; Wang et al., 2022). The capacity of TAMs to penetrate into and persist in solid malignancies might not only be crucial for targeted CAR-M treatment in LC, but it could also be utilized for organoid immunotherapy testing. A recent study by Dekkers et al., 2023, details how single-cell imaging of engineered T cells with solid tumor PDOs could prove useful in establishing the extent of their immunotherapeutic potential. Their newly developed cell tracking system enabled them to track and pinpoint engineered T cells with a particularly potent tumor-killing capacity (Dekkers et al., 2023). The emergence of such methods might be further applied for the evaluation of CAR-M efficacy against LC PDOs in the future. Nowadays, patient-matched healthy and malignant LC PDOs can be easily attained in order to compare the effects of CAR-M delivery to healthy versus cancer tissues. iPSC-derived CAR-Ms could be particularly useful for such experimentation, as their ex vivo expansion potential is unlimited (Zhang et al., 2020).

Breakthroughs involving organoid development are being paralleled by the evolution of platforms replicating the TME via organoid-immune cell co-cultures, such as lung-on-a-chip. This biomimetic approach could eventually be used to model the contribution of a greater range of TME cells toward NSCLC, providing a complex overview of the pro- and antitumorigenic signaling interplay within the TME. To conclude, the utility of complex 3D co-culture systems is supported by an increasingly available array of methods that enable the investigation of the tumor cell-immune cell interactions. In the future, organoid-macrophage co-culture platforms might have promising applications for NSCLC disease modeling, pre-clinical immunotherapeutic testing, as well as personalized medicine devoid of reliance on the use of animal models.
